# Expanding and exploring the diversity of phytoplasmas from lucerne (*Medicago sativa*)

**DOI:** 10.1038/srep37746

**Published:** 2016-11-25

**Authors:** David Gopurenko, Murray J. Fletcher, Jian Liu, Geoff M. Gurr

**Affiliations:** 1 Graham Centre for Agricultural Innovation (Charles Sturt University & NSW Department of Primary Industries), NSW Department of Primary Industries, Wagga Wagga Agricultural Institute, Pine Gully Rd, Wagga Wagga, NSW 2650, Australia; 2 Graham Centre for Agricultural Innovation (Charles Sturt University & NSW Department of Primary Industries), PO Box 883, Orange, NSW 2800, Australia; 3 State Key Laboratory of Ecological Pest Control for Fujian and Taiwan Crops, Fujian Agriculture and Forestry University, Fuzhou 350002, China; 4 Institute of Applied Ecology, Fujian Agriculture & Forestry University, Fuzhou 350002, China

## Abstract

Phytoplasmas are a group of insect-vectored bacteria responsible for disease in many plant species worldwide. Among the crop species affected is the economically valuable forage species lucerne. Here we provide comprehensive molecular evidence for infection in multiple lucerne plants by a phytoplasma not previously known from this plant species. This phytoplasma had a >99% genetic similarity to an unclassified 16S rRNA subgroup previously reported as Stylosanthes little leaf from *Stylosanthes* spp. and was genetically and symptomatically distinct from a co-occurring but less common 16SrIIA group phytoplasma. Neighbour-joining analyses with publicly available sequence data confirmed the presence of two distinct phytoplasma lineages in the plant population. No PCR detections were made among 38 individuals of 12 co-occurring weed species. Sequence analysis revealed that all nine PCR detections from among 106 individuals of five Hemiptera insect species from the site, three of which had previously been reported as likely vectors, were false positives. This study demonstrates the importance of sequencing to complement PCR detection and avoid potentially inaccurate conclusions regarding vectors, highlights that sampling over a wide spatio-temporal scale is important for vector and alternative host studies, and extends to eight the number of phytoplasma 16 Sr groups known from lucerne.

The ‘*Candidatus* Phytoplasma’ taxon (trivial name: phytoplasma) comprises a group of wall-less, non-helical prokaryotes that are unusual in being able to invade and reproduce within both living plants and insects^1^. They are associated with a broad variety of plant pathologies and are generally vectored among host plants by sap feeding insects in the order Hemiptera. Plant symptoms of phytoplasma infection include chlorosis, stunting and proliferation of shoots giving rise to disease common names such as yellowing, little leaf and witches’ broom. Molecular sequence studies of phytoplasmas indicate the group constitutes a genetically distinct and coherent taxon with a rich diversity of groups and subgroups^2^. Protocols for formal genetic species description within this taxon are based on comparisons of 16S rRNA-encoding gene sequences. Formal recognition of bacteria species in the class Mollicutes requires description of cultured growth activity which is not observable with phytoplasmas. Accordingly, a new ‘species’ is assigned if it has <97.5% sequence similarity to existing described taxa^1^. Based on this system, 37 formally described candidate species of ‘*Ca*. Phytoplasma’ and more than 10 putatively novel species have been reported^1^,^3^ and classified to 33 RFLP based 16 S rRNA subgroups (and others undetermined) associated with various phytoplasma-induced plant diseases^4^. The list of formally described ‘*Ca*. phytoplasma’ species is likely to increase as research examines a broader variety of pathological symptoms in emerging crop industries^5^,^6^,^7^ and by improved genetic species resolution using alternative gene targets^8^,^9^.

The legume plant lucerne (*Medicago sativa* L), also known as alfalfa, is commercially grown as a pasture, hay and seed crop in many locations worldwide and suffers from several serious diseases with phytoplasma aetiology. The most internationally prevalent is Alfalfa witches’ broom (AWB)^10^. The stolbur phytoplasma has been reported from lucerne in Italy^11^ and Serbia^12^, littleleaf phytoplasma from India^13^ and aster yellows phytoplasma from the USA^14^, as well as being implicated as a reservoir for canola yellows phytoplasma^15^. Because these have long been known by common disease symptom names, and 16S rRNA subgroups are a more recent advance in nomenclature, there is no clear cut correspondence between these names and subgroups. Based on publicly available sequence accessions at GenBank (ncbi.nlm.nih.gov), seven 16 Sr phytoplasma groups (I, II, III, V, VI, VII, XII) are known to affect lucerne (Fig. 1). In Australia, the first report of a phytoplasma associated with lucerne was detection of sweet potato little leaf strainV4 (SPLL-V4), a 16SrII group phytoplasma, in a single specimen exhibiting little leaf symptoms from the Northern Territory^16^. A study of Australian lucerne yellows (AluY) disease, which has symoptoms distinct from little leaf^17^, used electron microscopy to visualise phytoplasma bodies in symptomatic plants and initial analysis of 16S rRNA revealed this strain to be closely related phylogenetically to Australian tomato big bud phytoplasma^18^, a 16SrII group phytoplasma^8^. More comprehensive study identified the phytoplasma associated with ALuY disease to be within the faba bean phyllody phytoplasma 16SrII group^19^. Later work on Australian lucerne yellows disease reported a second phytoplasma, 16SrXII-B subgroup strain from lucerne in New South Wales^20^. Over the intervening decade there has been no further report of phytoplasmas from lucerne in Australia, meaning that only two of the seven internationally reported 16Sr groups are known. It is important to establish a more complete understanding of this to inform biosecurity, most particularly the possibility of incursions by additional exotic stains. More generally, there is a lack of integrative studies of the complex of phytoplasma types that affect lucerne globally.

Knowledge of a pathogen, including its genetic diversity, is fundamental to epidemiological understanding and rational management practices. In the case of phytoplasmas, information on the insects capable of vectoring the pathogen, and on any weed species that may constitute alternative hosts, is also key. Accordingly, this study aimed to study the genetic diversity of phytoplasmas from a population of lucerne plants in a single field and to place this into a global context. To maximise the chances of detection, tissue samples were taken from plants exhibiting chlorosis or witches’ broom symptoms, whilst corresponding samples were also taken from the nearest healthy neighbouring lucerne plant, as well as from 38 weeds of 12 species growing within the lucerne field. A total of 106 individual Hemiptera insects that were potential vectors were also sampled from the field. All samples were processed in the laboratory using relevant molecular techniques to identify the insects and to detect and characterise phytoplasmas.

## Results

### PCR detection of putative phytoplasmas

Serial PCR using 2nd stage primers fU5 and m23sr tested positive for putative phytoplasma presence in nine lucerne plants. These nine plants displayed outward symptoms of phytoplasma infection and also tested positive in serial PCR using an alternative 2nd stage primer set 16r758F and m23sr (Table 1). Two additional lucerne plants (one of these symptomless) tested positive using this alternative primer set. Nine of the insects tested PCR positive (Table 1), two using primer set fU5 & m23sr and nine using 16r758F & m23sr. None of the weeds tested PCR positive using either primer set.

### Sequence analysis of putative phytoplasma positives

Sequence queries at GenBank using BLAST confirmed phytoplasma identities for the nine lucerne samples shown to be PCR positive using 2nd stage primer set fU5 & m23sr, and alternative primer set 16r758F and m23sr. Sequences of the two additional PCR positive lucerne samples detected using the alternative primer set were matched to bacteria other than phytoplasma (Supplementary Table S1). PCR positives detected for all nine insects were false positives, matching a variety of non-phytoplasma bacteria (Supplementary Table S2). Across all plant and insect samples, 11 and 2 PCR positives matched bacteria other than phytoplasma using primer set 16r758F & m23sr, and fU5 & m23sr respectively.

Of the nine sequence-confirmed positive infected plants, two displaying witches’ broom symptoms had an identical sequence (GenBank accession KX421797) match (>99.8% similarity) to 16SrII-A subgroup sequences for a variety of near identical strain accessions including JQ067649 (Sweet potato little leaf phytoplasma [SPLL]) found in Australia, and less similar (>98%) to other 16SrII subgroup phytoplasmas infecting lucerne in Australia and elsewhere. The remaining seven confirmed phytoplasma positive plants had four nearly identical sequences (GenBank accessions KX421793- KX421796) matched (>99.9% similarity) to two accessions AJ289192 and Y17055 reported for Stylosanthes little leaf [StLL] phytoplasma, currently unplaced to a 16S rRNA subgroup. These seven lucerne plants exhibited uniform symptoms of yellowing and stunting of leaves, consistent with StLL infection^21^.

### Phylogenetic analysis

**Figure 1:**
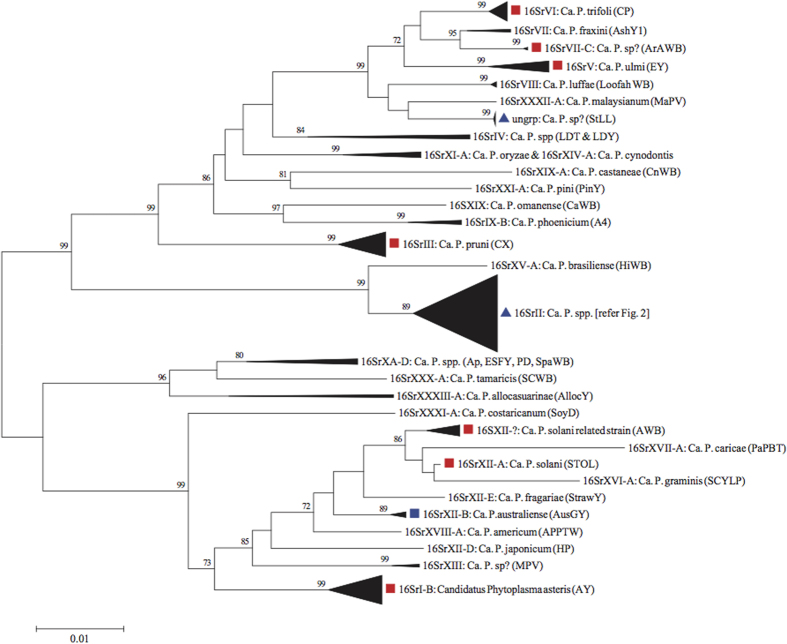
Neighbour-joining distance tree of 16S rRNA phytoplasma sequence accessions (N = 163) and sequences of nine phytoplasma-positive lucernes from Forbes, Australia. Scale bar equals 1% equal weighted sequence difference. Cluster node supports >70% (10,000 bootstrap replicates) as indicated. Terminal 16S rRNA subgroups (refer Methods) collapsed as clusters containing multiple accessions. Tip labels indicate 16S rRNA subgroups, provisional *Candidatus* Phytoplasma species, and (in parentheses) associated phytoplasma strain or disease acronym. Multiple provisional species in clusters indicated as “spp.”, unknown species as “sp?”. Shaded red squares, blue squares, and blue triangles indicate phytoplasma detected in lucerne overseas, Australia and Forbes respectively. Refer Fig. 2 for greater detail of the 16SrII subgroup cluster.

**Figure 2:**
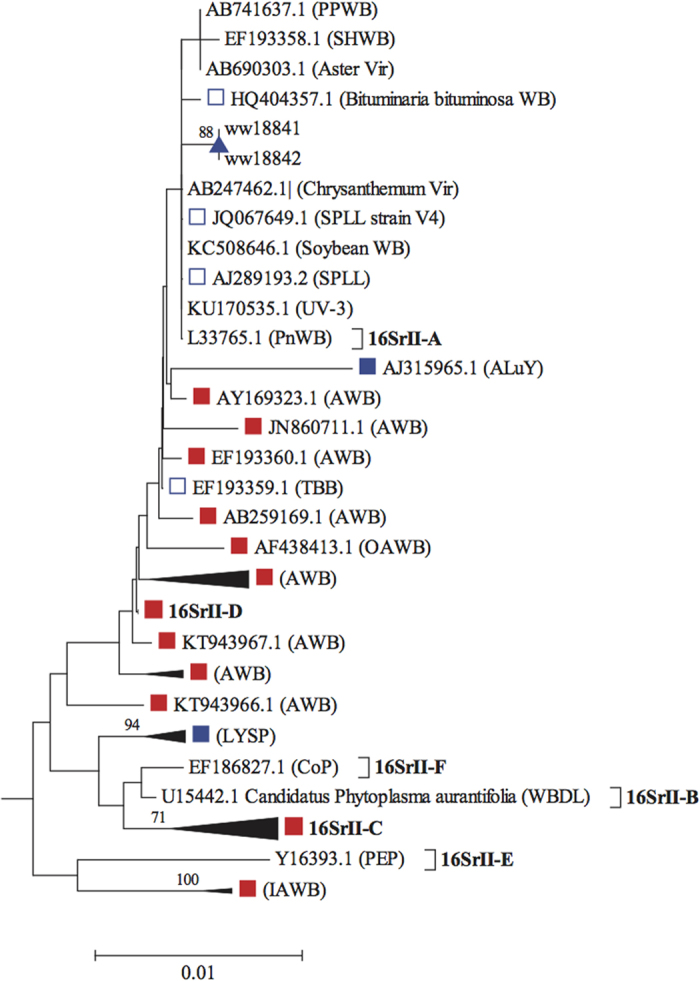
Neighbour-joining distance tree of sequence accessions (N = 50) and two Forbes lucerne samples (ww18841 & ww18842), in “***Candidatus*** Phytoplasma aurantifolia” 16SrII subgroup cluster from Fig. 1. Scale bar, cluster supports and shaded shapes as per Fig. 1; unshaded blue squares indicate phytoplasma in Australian plants other than lucerne. Tip labels indicate GenBank accession # (except where multiple accessions are collapsed), and (in parentheses) associated phytoplasma strain or disease acronyms. Tips/clusters containing accessions identified to 16SrII subgroups A to F^44^ as indicated in bold.

Neighbour-joining analysis (Fig. 1 & Supplementary Figure 1) confirmed presence of two distinct genetic lineages of phytoplasma differing by ~9.76% among the lucerne positives. Two specimens (ww18841, ww18842) were nested in the “*Ca*. P. aurantifolia” species cluster containing 16SrII-A subgroup phytoplasma strains associated with a variety of diseases and plant hosts (Fig. 2). The two specimens more closely matched sequences in 16SrII-A subgroup (associated with multiple strains of witches’ broom and virescence in several host plants), than to other 16SrII subgroup strains accessioned to lucerne, including LYSP-E2 (accession JX861231), ALuY (accession AJ315965^19^) strain in Australia, and a variety of other lucerne disease strains in the Middle East and Europe.

The 2nd phytoplasma lineage detected was from seven lucerne plants and clustered with an undescribed 16S rRNA subgroup previously identified to Stylosanthes little leaf phytoplasma (StLL) previously reported from *Stylosanthes* legumes^21^,^22^,^23^. StLL is unusual among phytoplasmas in containing two independent 16S-23S rRNA operons, one lacking the internal tRNA^Ile^ gene that is normally present and characteristic in all other phytoplasmas^21^. Absence of this tRNA^Ile^ gene from a phytoplasma 16S-23S rRNA-encoding operon has not been reported elsewhere. Sequences here identified both presence and absence of the tRNA^Ile^ gene among samples identified to the StLL lineage, in agreement with prior reports for this phytoplasma.

Phytoplasma subgroup 16SrXII-B strain previously reported infecting NSW lucerne^20^ and seven other 16 S rRNA subgroups infecting lucerne outside of Australia (Fig. 1), were not evident among the samples tested in this study.

### DNA barcode identification of insects

CO1 ‘DNA barcode’ sequences were obtained from 95 insects. Sequence queries at BOLD (Supplementary Table S2) identified 82 insects with >98% genetic similarity to curated voucher specimens of Hemiptera species, including positive identifications to leafhoppers *Austroagallia torrida* Evans (N = 78), *Orosius argentatus* (Evans) (N = 1), *Orosius orientalis* (Matsumura) (N = 2) and a mirid bug *Campylomma* sp. (N=1). The remaining 13 insects were matched (>99% sequence similarity) to a unidentified Typhlocybinae species present in South Australia and Queensland. Taxonomic examination of these 13 specimens placed them as an undetermined species of *Austroasca* leafhopper (Typhlocybinae: Empoascini).

## Discussion

Several genetically distant 16Sr groups of phytoplasma have been reported as the potential etiological agents leading to disease symptoms in lucerne described as “yellows” and “witches’ broom”. For example, in Australia, Lucerne yellows disease (ALuY), has been associated with separate phytoplasma 16S rRNA groups II^19^ and XII^20^, respectively. Worldwide, seven distinct 16S rRNA subgroups have been reported in lucerne symptomatic for yellows and witches broom related diseases. Cataloguing the diversity of phytoplasma sequence strains and 16Sr groups present in lucerne, their associations with disease symptoms and their host and vector arthropod specificity is a necessary primary step for evidence-based biosecurity measures and management.

Here we report molecular genetic evidence of two distinct phytoplasmas detected among lucerne plants. Lucerne symptomatic in the field for witches’ broom had >99.85% sequence similarity to various near identical 16S rRNA subgroup II-A strains associated with diseases in other, botanically unrelated crops including sweetpotato little leaf (SPLL-V4). 16SrII-A strains such as SPLL-V4 have been previously reported in lucerne sampled in Australia^16^,^24^. Other 16SrII subgroups ascribed to the *Ca*. P. aurantifolia species, contain a broad variety of sequence strains associated with lucerne diseases including Alfalfa witches broom (in the Middle East and Europe)^25^ and Australian lucerne yellows (ALuY & LYSP-E2)^26^,^27^; each of which is marginally less similar in sequence identity to the present study’s specimens than are the aforementioned 16SrII-A accessions.

Present evidence of 16SrII-A in lucerne, confirms broad host use by this subgroup of phytoplasma, reported previously from tomato and eggplant (Solanaceae), sweetpotato (Convolvulaceae), and four taxonomically diverse weeds (*Alysicarpus* sp. (Fabaceae), *Amaranthus* sp. (Amaranthaceae), *Passiflora foetida* (Passifloraceae) and *Evolvulus* sp. (Convolvulaceae) in northern Australia^28^. *Amaranthus* was noted by Gibb, *et al*.^28^ as potentially of epidemiological significance for spread of this phytoplasma to crops in northern Australia because it grows in close association with sweetpotato and supports high levels of the supposed vector insect *O. argentatus*. Fletcher, *et al*.^29^ provide evidence that *O. argentatus* is a valid species and distinct from *O. orientalis* with which it had previously been synonymised by Kwon and Lee^30^.

The other phytoplasma strain detected in the present study was associated with symptoms of yellowed/ stunted leaves in lucerne plants, and genetically identified (>99.9% sequence similarity) to the ungrouped but phylogenetically unique Stylosanthes little leaf [StLL] phytoplasma previously identified in *Stylosanthes* legumes^23^. The discovery here of the StLL phytoplasma present in lucerne raises the number of 16Sr groups reported from this important crop to three within Australia and to eight globally. Significantly, lucerne and other legumes in the genus *Stylosanthes* share at least two disparate 16S rRNA subgroups indicating the two legume genera have shared susceptibility to distantly related phytoplasmas. It remains to be determined if the plants are also hosts to a common assemblage of vector insects infective for the two phytoplasma subgroups.

In our survey, phytoplama presence was not detected among any of the phloem feeding insects sampled in the vicinity of the infected lucerne. This is surprising given that our insect sample was dominated by presence of *A. torrida*, a leafhopper species previously identified as a potential vector of lucerne phytoplasma^31^, and a lesser number of other putative vector species (*O. argentatus* and *O. orientalis*). The presence of false positives among the insects suggests that there were no deficiencies in sample preparation and processing; rather that phytoplasmas were absent. This indicates that the collection of Hemiptera species (even those present in large numbers) from host plants that are proven to be infected by phytoplasmas (two diverse strains) is not evidence for vector status. Clearly sampling over a larger spatio-temporal scale will be necessary to establish which insect species may test PCR positive though transmission tests are necessary to provide definitive evidence of vector capacity^32^.

False positives were observed in each of the two independent serial PCR assays using different forward primers in 2nd stage PCR. The effect was more prevalent when primer 16r758F was used as an alternative forward primer to fU5 in 2nd stage PCR. Optimization of PCR annealing temperature above that used here may increase targeted stringency to phytoplasma amplification and eliminate false positive presence, but also potentially result in increased frequency of false PCR negatives in instances where there is nucleotide variation among phytoplasma strains at primer annealing sites. Regardless, the presence of false positives in serial PCR indicates sequencing is a necessary requisite for confirming phytoplasma presence and identity when PCR positives are detected. Alternatives to sequencing such as restriction digest profiling of PCR positives may be expedient for confirming presence of phytoplasma and even subgroups of phytoplasma, but has limited capability for detection of novel phytoplasma varieties.

## Methods

### Sampling

Leaf samples from 64 plants from an agricultural field site at Forbes New South Wales (NSW), Australia (−33.381S, 147.976 E) sampled Feburary 2013 were genetically tested for phytoplasma presence and identity (Table 1 and Supplementary Table S1). Samples included lucernes (N = 14) and weeds (N = 6) with witches’ broom symptoms of leaf chlorosis, stunting and/or leaf bunching. Neighbouring asymptomatic lucernes (N = 12) and various weeds (N = 32) were also sampled, as were phloem feeding Hemiptera (N = 106) captured using sweep nets. Specimens were individually catalogued with unique specimen ID labels, preserved in >70% ethanol, and curated at NSW Department of Primary Industries agricultural institutes in Orange (insects) and Wagga Wagga (plants).

### Non-destructive DNA extraction

DNA extraction was preceded by a non-destructive tissue digestion. Whole insects, and plant leaf laminar (<0.2 g), were individually placed in 480 μL aliquots of DXT tissue digestion buffer (QIAGEN, Doncaster, Australia) incorporating 1% DX digestion buffer additive (QIAGEN) and digested overnight at 55 °C. Specimens were later removed from the digests and stored in 70% ethanol. DNA was extracted from 240 μL of each specimen digest using a Corbett Research 1820 X-tractor Gene robotic system and associated DNA extraction kit reagents (QIAGEN). DNA was eluted to 150 μL and stored at −20 °C.

### PCR amplification of target genes and sequencing

Polymerase chain reaction (PCR) amplifications were prepared to final reaction volumes of 15 μL using a Corbett CAS-1200 automated liquid handling workstation. Reactions included 1 μL of DNA extract in the presence of Invitrogen ^TM^ reagents: 1x buffer, 2.8 mM MgCl_2_, 0.4 units of Platinum^®^ Taq DNA polymerase, 200 μM dNTPs and 2 pmol of forward and reverse primers. PCR cycling used an Eppendorf Mastercycler *ep gradient S* machine set to the thermal profile: 94 °C for 2 min; 40 cycles of 94 °C for 30 s, 50 °C for 30 s, 72 °C for 1 min; 72 °C for 5 min; final 4 °C.

PCR for DNA barcoding^33^ of insects targeted a 667 base pair (bp) portion of the 5′ mitochondrial cytochrome *c* oxidase I (COI) gene using primers described in Fletcher, *et al*.^29^. PCR products were visualized by UV trans-illumination after electrophoresis through a 1.5% agarose gel in 1% TAE buffer containing SYBR^®^ Safe DNA gel stain (Invitrogen^TM^), and qualitatively checked for expected fragment size against E-Gel size marker (Invitrogen^TM^). PCR products were sent to the Australian Genome Research Facility (Brisbane) for purification and bidirectional sequencing using an Applied Biosystems 3730*xl* DNA Analyzer.

PCR for positive/negative detection of infective phytoplasma genes in host plants and insects targeted amplification of the partial 16S–23S nuclear ribosomal DNA gene region (16S–23 S rRNA) containing the complete tRNA^Ile^ gene and surrounding intergenic spacer regions. A two-stage serial PCR procedure modified from Pilkington, *et al*.^19^ was used to enhance amplification of phytoplasma gene targets, given the expected low titre of bacterial DNA in host specimen extracts^34^. The 1st stage PCR amplified >1.8-kbp product using primers P1^35^ and P7^36^. 1st stage PCR products were diluted (1:100) and used as templates in 2nd stage PCR with primers fU5^37^ and m23Sr^38^ to amplify an internal >1.4-kbp product. 2nd stage PCR was repeated substituting primer 16r758F^28^ for primer fU5, to amplify a smaller portion (>1.0-kbp) of the 16S-23S rRNA region. PCR reactions were prepared as described earlier for DNA barcoding with the exceptions of primers used, and an increased PCR annealing temperature set at 55 °C. 2nd stage PCR products were visualized after electrophoresis as described earlier, and positives in expected size ranges were sequenced (as described earlier). In several instances dual PCR products in the expected range, but separated by approximately 100 bp, were observed in individual specimen PCR. In these instances, 2nd stage PCR products were re-run through a 1% agarose gel in 1% TBE buffer to allow size separation of the dual products. Size separated products were excised from the gel and individually purified of agarose using QIAquick Gel Extraction Kit (QIAGEN) prior to their independent sequencing.

### Sequence analyses

Forward and reverse sequence chromatograms were assembled to specimen ID, primer truncated and checked for signal quality using Lasergene SeqMan Pro ver. 8.1.0(3) (DNASTAR Inc., Maddison, WI, USA). Phytoplasma PCR positive sequences were re-aligned for indel positions using MUSCLE^39^ implemented in *MEGA* version 6^40^.

Insect DNA barcode sequences were queried for species identity (20 June 2016) at the Barcode of Life Data systems^41^ online sequence repository. BOLD specimen sequences with >98% sequence similarity to our query sequences were considered conspecific. Insect specimen records and DNA barcode sequences are available as a BOLD dataset (http://dx.doi.org/10.5883/DS-AUHEMI01).

Sequences of phytoplasma-positive PCRs were queried for identity against GenBank (and EMBL) sequence accessions using the online NCBI BLAST tool. 16S rRNA phytoplasma accessions matched at >99% similarity and coverage to query sequences were included in an alignment containing accessions (Supplementary Table S3) representative of 16S rRNA subgroups and 37 provisionally identified ‘*Candidatus* Phytoplasma’ species^42^, and accessions reported in *Medicago sativa* (lucerne, alfalfa). The alignment (N = 172) was truncated to 1171nt (including gaps) corresponding to positions 421–1527 of accession FJ943262 (*Candidatus* Phytoplasma australiense strain NZ09156). Pair-wise % genetic distances (sites equally weighted; missing sites and indels excluded) between sequences were compared as a neighbor-joining (NJ) tree^43^ using *MEGA* version 6^40^. Support values for NJ clusters were estimated by non-parametric bootstrapping (10 000 replicates). Representative phytoplasma sequences from field collected specimens were deposited at GenBank under accession records KX421793-KX421797.

## Additional Information

**How to cite this article**: Gopurenko, D. *et al*. Expanding and exploring the diversity of phytoplasmas from lucerne (*Medicago sativa*). *Sci. Rep.* **6**, 37746; doi: 10.1038/srep37746 (2016).

**Publisher's note:** Springer Nature remains neutral with regard to jurisdictional claims in published maps and institutional affiliations.

## Supplementary Material

Supplementary Information

## Figures and Tables

**Table 1:** Summary of specimen sampling and phytoplasma testing in lucerne, weeds and insects sampled from an agricultural field site in Forbes, NSW.

Sample	*N*	*N*_symp_	*N*_PCR+_	*N*_seq+_	16Sr group
Lucerne	26	14	11	9	II-A & ungrouped StLL
Weeds (12 spp)	38	6	0	0	—
Insects (5 spp)	106	—	9	0	—

Number (*N*) of sampled specimens symptomatic (*N*_symp_), PCR positive (*N*_PCR+_), and sequence confirmed (*N*_seq+_), for phytoplasma infection as indicated. 16Sr groups as indicated.

## References

[b1] IRPCM. ‘*Candidatus* Phytoplasma’, a taxon for the wall-less, non-helical prokaryotes that colonize plant phloem and insects. Int. J. Syst. Evol. Microbiol. 54, 1243–1255, doi: 10.1099/ijs.0.02854-0 (2004). 15280299

[b2] Lee I.-M., Gundersen-Rindal D. E., Davis R. E. & Bartoszyk I. M. Revised Classification Scheme of Phytoplasmas based on RFLP Analyses of 16S rRNA and Ribosomal Protein Gene Sequences. International Journal of Systematic Bacteriology 48, 1153–1169, doi: 10.1099/00207713-48-4-1153 (1998). 9542097

[b3] Harrison N. . ‘*Candidatus* Phytoplasma palmicola’, a novel taxon associated with a lethal yellowing-type disease (LYD) of coconut (*Cocos nucifera* L.) in Mozambique. Int. J. Syst. Evol. Microbiol. 64, doi: 10.1099/ijs.0.060053-0 (2014). 24585372

[b4] Davis R. E. *‘Candidatus Phytoplasma’ species*, http://plantpathology.ba.ars.usda.gov/pclass/pclass_phytoplasmaclassification_system2.html (2016). Molecular Plant Pathology Laboratory (MPPL). Date of access: 26 July 2016.

[b5] Wei W., Davis R. E., Lee I.-M. & Zhao Y. Computer-simulated RFLP analysis of 16S rRNA genes: identification of ten new phytoplasma groups. Int. J. Syst. Evol. Microbiol. 57, 1855–1867, doi: 10.1099/ijs.0.65000-0 (2007). 17684271

[b6] Pérez-López E., Luna-Rodríguez M., Olivier C. Y. & Dumonceaux T. J. The underestimated diversity of phytoplasmas in Latin America. Int. J. Syst. Evol. Microbiol. 66, 492–513 (2016). 26519050 10.1099/ijsem.0.000726

[b7] Davis R. I. . A new wilt disease of banana plants associated with phytoplasmas in Papua New Guinea (PNG). Australasian Plant Dis. Notes 7, 91–97, doi: 10.1007/s13314-012-0056-8 (2012).

[b8] Hodgetts J., Boonham N., Mumford R., Harrison N. & Dickinson M. Phytoplasma phylogenetics based on analysis of secA and 23S rRNA gene sequences for improved resolution of candidate species of ‘*Candidatus* Phytoplasma’. Int. J. Syst. Evol. Microbiol. 58, 1826–1837, doi: 10.1099/ijs.0.65668-0 (2008). 18676464

[b9] Duduk B. & Bertaccini A. Phytoplasma classification: Taxonomy based on 16S ribosomal gene, is it enough? Phytopathogenic Mollicutes 1, 3–13 (2011).

[b10] Khan A. J., Botti S., Al-Subhi A. M., Gundersen-Rindal D. E. & Bertaccini A. F. Molecular Identification of a New Phytoplasma Associated with Alfalfa Witches’-Broom in Oman. Phytopathology 92, 1038–1047, doi: 10.1094/PHYTO.2002.92.10.1038 (2002). 18944213

[b11] Marzachì C. . Molecular hybridization and PCR amplification of nonribosomal DNA to detect and differentiate stolbur phytoplasma isolates from Italy. J. Plant Pathol. 82, 201–212 (2000).

[b12] Starović M. . Detection and identification of two phytoplasmas (16SrIII‐B and 16SrXII‐A) from alfalfa (*Medicago sativa*) in Serbia. J. Phytopathol. 160, 758–760 (2012).

[b13] Suryanarayana V., Singh S., Muniyappa V. & Reddy H. Little leaf of *Medicago sativa* L.-A new phytoplasma disease in India. Int. J. Trop. Plant Dis. 14, 167–171 (1996).

[b14] Peters R. D. . First Report of Aster Yellows Phytoplasma in Alfalfa. Plant Dis. 83, 488–488, doi: 10.1094/PDIS.1999.83.5.488C (1999). 30845553

[b15] Wang K. & Hiruki C. Use of Heteroduplex Mobility Assay for Identification and Differentiation of Phytoplasmas in the Aster Yellows Group and the Clover Proliferation Group. Phytopathology 91, 546–552, doi: 10.1094/PHYTO.2001.91.6.546 (2001). 18943942

[b16] Wilson D., Blanche K. R. & Gibb K. S. Phytoplasmas and disease symptoms of crops and weeds in the semi-arid tropics of the Northern Territory, Australia. Australas. Plant Path. 30, 159–163, doi: 10.1071/AP01015 (2001).

[b17] Pilkington L., Gurr G. M., Fletcher M. J., Nikandrow A. & Elliott E. Occurrence and severity of lucerne yellows disease in Australian lucerne seed crops. Australas. Plant Path. 28, 235–239 (1999).

[b18] Pilkington L. J. . First report of a phytoplasma associated with Australian lucerne yellows disease. Plant Pathol. 51, 390, doi: 10.1046/j.1365-3059.2002.00703.x (2002).

[b19] Pilkington L. J. . Detection and identification of a phytoplasma from lucerne with Australian lucerne yellows disease. Plant Pathol. 52, 754–762, doi: 10.1111/j.1365-3059.2003.00934.x (2003).

[b20] Getachew M. A. . First report of a “*Candidatus* Phytoplasma australiense”-Related strain in lucerne (*Medicago sativa*) in Australia. Plant Dis. 91, 111, doi: 10.1094/pd-91-0111a (2007). 30781081

[b21] De La Rue S., Padovan A. & Gibb K. *Stylosanthes* is a Host for Several Phytoplasmas, One of which Shows Unique 16S-23S Intergenic Spacer Region Heterogeneity. J. Phytopathol. 149, 613–619, doi: 10.1046/j.1439-0434.2001.00683.x (2001).

[b22] Davis R., Schneider B. & Gibb K. Detection and differentiation of phytoplasmas in. Aust. J. Agric. Res. 48, 535–544 (1997).

[b23] Schneider B. . Detection and differentiation of phytoplasmas in Australia: an update. Aust. J. Agric. Res. 50, 333–342 (1999).

[b24] Gibb K., Mowles A. & Randles J. Mundulla Yellows Phytoplasma: Research Report. (University of Adelaide, Adelaide, Australia, 2000).

[b25] Khan A., Azam K., Deadman M., Al-Subhi A. & Jones P. First Report of Alfalfa Witches Broom Disease in Oman Caused by a Phytoplasma of the 16Sr II Group. Plant Dis. 85, 1287–1287 (2001). 10.1094/PDIS.2001.85.12.1287A 30831799

[b26] Streten C. & Gibb K. S. Phytoplasma diseases in sub-tropical and tropical Australia. Australas. Plant Path. 35, 129–146, doi: 10.1071/AP06004 (2006).

[b27] Yang S. . Three group 16SrII phytoplasma variants detected in co-located pigeonpea, lucerne and tree medic in South Australia. Australasian Plant Dis. Notes 8, 125–129 (2013).

[b28] Gibb K., Padovan A. & Mogen B. Studies on sweet potato little-leaf phytoplasma detected in sweet potato and other plant species growing in Northern Australia. Phytopathology 85, 169–174 (1995).

[b29] Fletcher M., Löcker H., Mitchell A. & Gopurenko D. A revision of the genus *Orosius* Distant (Hemiptera: Cicadellidae: Deltocephalinae) based on male genitalia and DNA barcoding. *Austral Entomology*, doi: 10.1111/aen.12224.

[b30] Kwon Y. & Lee C. On some new and little known Palaearctic species of leafhoppers (Homoptera: Auchenorrhyncha: Cicadellidae). Nature and Life 9, 69–97 (1979).

[b31] Pilkington L. J., Gurr G. M., Fletcher M. J., Nikandrow A. & Elliott E. Vector status of three leafhopper species for Australian lucerne yellows phytoplasma. Aust. J. Entomol. 43, 366–373, doi: 10.1111/j.1440-6055.2004.00419.x (2004).

[b32] Gurr G. M., Ash G. J., Wilson B., Ero M., Pilotti C., Dewhurst C. & You Y. Coconut lethal yellowing diseases: a global threat to palms of economic and social significance. Frontiers in Plant Science 7, doi: 10.3389/fpls.2016.01521. PMC5080360 27833616

[b33] Hebert P. D., Cywinska A. & Ball S. L. Biological identifications through DNA barcodes. Proceedings of the Royal Society of London B: Biological Sciences 270, 313–321 (2003). 10.1098/rspb.2002.2218 PMC1691236 12614582

[b34] Bertaccini A. . Sensitive detection of mycoplasmalike organisms in field-collected and *in vitro* propagated plants of *Brassica, Hydrangea* and *Chrysanthemum* by polymerase chain reaction. Ann. Appl. Biol. 121, 593–599, doi: 10.1111/j.1744-7348.1992.tb03469.x (1992).

[b35] Deng S. & Hiruki C. Amplification of 16S rRNA genes from culturable and nonculturable Mollicutes. Journal of Microbiological Methods 14, 53–61, doi: 10.1016/0167-7012(91)90007-D (1991).

[b36] Kirkpatrick B. . Phylogenetic relationships of plant pathogenic MLOs established by 16/23S rDNA spacer sequences. IOM Lett 3, 228–229 (1994).

[b37] Lorenz K., Schneider B., Ahrens U. & Seemüller E. Detection of the apple proliferation and pear decline phytoplasmas by PCR amplification of ribosomal and nonribosomal DNA. Phytopathology 85, 771–776 (1995).

[b38] Padovan A. C. . Molecular detection of the Australian grapevine yellows phytoplasma and comparison with grapevine yellows phytoplasmas from Italy. Australian Journal of Grape and Wine Research 1, 25–31, doi: 10.1111/j.1755-0238.1995.tb00074.x (1995).

[b39] Edgar R. C. MUSCLE: multiple sequence alignment with high accuracy and high throughput. Nucleic Acids Research 32, 1792–1797, doi: 10.1093/nar/gkh340 (2004). 15034147 PMC390337

[b40] Tamura K., Stecher G., Peterson D., Filipski A. & Kumar S. MEGA6: Molecular Evolutionary Genetics Analysis Version 6.0. Mol. Bio. Evol. 30, 2725–2729, doi: 10.1093/molbev/mst197 (2013). 24132122 PMC3840312

[b41] Ratnasingham S. & Hebert P. D. BOLD: The Barcode of Life Data System. Mol. Ecol. Notes 7, 355–364 (2007). 18784790 10.1111/j.1471-8286.2007.01678.x PMC1890991

[b42] Gurr G. M. . In Sustainable Pest Management in Date Palm: Current Status and Emerging Challenges (eds Waqas Wakil, Romeno Faleiro Jose & Miller Thomas A.) 287–314 (Springer International Publishing, 2015).

[b43] Saitou N. & Nei M. The neighbor-joining method: a new method for reconstructing phylogenetic trees. Mol. Bio. Ev 4, 406–425 (1987). 10.1093/oxfordjournals.molbev.a040454 3447015

[b44] Fránová J., Ludvíková H., Paprštein F. & Bertaccini A. Genetic diversity of Czech ‘*Candidatus* Phytoplasma mali’ strains based on multilocus gene analyses. Eur. J. Plant Pathol. 136, 675–688, doi: 10.1007/s10658-013-0196-5 (2013).

