# Supplementary material for: Expanding and exploring the diversity of phytoplasmas from lucerne (Medicago sativa)
Source: Sci Rep. 2016 Nov 25;6:37746. doi: 10.1038/srep37746 (PMC5123570; doi:10.1038/srep37746)
Supplement: Supplementary Information [file srep37746-s1.pdf]

Supplementary Material

Expanding and exploring the diversity of phytoplasmas from lucerne (*Medicago sativa*)

David Gopurenko[1], Murray J Fletcher[2], Jian Liu[3,4,2] & Geoff M Gurr[3,4,2,*]

[1] NSW Department of Primary Industries, Wagga Wagga Agricultural Institute, Pine Gully Rd, Wagga Wagga, NSW, 2650

[2] Graham Centre for Agricultural Innovation (Charles Sturt University & NSW Department of Primary Industries), PO Box 883, Orange, NSW 2800

[3] State Key Laboratory of Ecological Pest Control for Fujian and Taiwan Crops, Fujian Agriculture and Forestry University, Fuzhou 350002, China

[4] Institute of Applied Ecology, Fujian Agriculture & Forestry University, Fuzhou 350002, China.

*Correspondence to: ggurr@csu.edu.au

Supplementary Table S1: Lucerne (*Medicago sativa*) and others plants sampled from Forbes (NSW) and tested for phtyoplasma. Symptoms of possible infection observed in the field. Phytoplasma positive (+) specimens identified in final stage serial PCR using primer pairs (16F758F & M23Sr) or (fU5 & M23Sr). BLAST queries of PCR + sequences at GenBank, closest matching 16SrDNA sequence accession, percentage (%) similarity and identification.

Specimens				PCR		16SrDNA query BLAST at GenBank		
sample ID	field label	Species	symptoms	16F758F-M23Sr	fU5-M23Sr	accession	%	identification
ww18808	206	*Chenopod* sp. 1						
ww18818	260	*Chenopod* sp. 1						
ww18821	269	*Chenopod* sp. 1						
ww18846	379	*Chenopod* sp. 1						
ww18880	463	*Chenopod* sp. 1						
ww18835	313	*Chenopod* sp.2						
ww18856	423	*Chenopod* sp.2						
ww18812	212	*Chenopod* sp.3						
ww18845	374	*Chenopod* sp.3						
ww18847	380	*Chenopod* sp.3						
ww18862	448	*Chenopod* sp.3						
ww18820	266	*Echium* sp.	distorted leaves					
ww18852	406	*Echium* sp.	distorted leaves					
ww18832	306	*Erigeron* sp.						
ww18840	350	*Erigeron* sp.						
ww18853	412	*Erigeron* sp.						
ww18866	453	*Erigeron* sp.						
ww18843	353	*Malva* sp.	yellows					

ww18851	404	*Malva* sp.	yellows					
ww18811	207	*Marrubium* sp.						
ww18830	298	*Marrubium* sp.						
ww18857	427	*Marrubium* sp.						
ww18864	450	*Marrubium* sp.						
ww18809	481	*Medicago sativa*						
ww18810	548	*Medicago sativa*						
ww18813	225	*Medicago sativa*						
ww18815	250	*Medicago sativa*	yellows & stunted leaves	+		Y17055	> 99	Phytoplasma StLL
ww18817	259	*Medicago sativa*						
ww18819	265	*Medicago sativa*						
ww18822	271	*Medicago sativa*	yellows & stunted leaves	+	+	Y17055	> 99	Phytoplasma StLL
ww18825	282	*Medicago sativa*						
ww18826	286	*Medicago sativa*	yellows					
ww18827	288	*Medicago sativa*		+		NR040809	100	Lactobacillales; *Weissella* sp.
ww18828	292	*Medicago sativa*						
ww18833	309	*Medicago sativa*						

ww18834	311	*Medicago sativa*	yellows	+	+	Y17055	> 99	Phytoplasma StLL
ww18837	332	*Medicago sativa*						
ww18838	346	*Medicago sativa*						
ww18839	349	*Medicago sativa*						
ww18841	351	*Medicago sativa*	WB		+	JQ067649	99	Phytoplasma SPLL
ww18842	352	*Medicago sativa*	WB	+	+	JQ067649	99	Phytoplasma SPLL
ww18850	403	*Medicago sativa*	yellows	+		HQ455821	> 99	Actinobacteria; *Arthrobacter globiformis*
ww18854	417	*Medicago sativa*	yellows					
ww18855	421	*Medicago sativa*	yellows	+	+	Y17055	> 99	Phytoplasma StLL
ww18859	432	*Medicago sativa*	yellows & stunted leaves	+	+	Y17055	> 99	Phytoplasma StLL
ww18861	443	*Medicago sativa*	yellows	+	+	Y17055	> 99	Phytoplasma StLL
ww18865	452	*Medicago sativa*	yellows & stunted leaves	+	+	AJ289192	> 99	Phytoplasma StLL
ww18882	484	*Medicago sativa*	yellows					
ww18885	498	*Medicago sativa*	yellows					
ww18823	274	*Onopordium* sp.						

ww18824	281	*Onopordium* sp.	
ww18849	395	*Onopordium* sp.	
ww18884	487	*Onopordium* sp.	
ww18814	243	*Polygonum* sp.	
ww18829	294	*Polygonum* sp.	
ww18848	386	*Polygonum* sp.	
ww18881	471	*Polygonum* sp.	
ww18831	301	*Solanacea* sp.	
ww18836	315	*Solanacea* sp.	
ww18844	366	*Tribulus* sp.	
ww18863	454	*Tribulus* sp.	
ww18816	256	Unidentified sp.	yellows
ww18858	430	Unidentified sp.	yellows
ww18860	433	Unidentified sp.	
ww18883	485	Unidentified sp.	

Supplementary Table S2: Hemiptera insects sampled from Forbes (NSW) and tested for phytoplasma DNA. Specimen records and DNA barcodes of each sample ID and available at BOLD as a dataset (http://dx.doi.org/10.5883/DS-AUHEMI01). Taxonomic identifications determined by morphological examination; DNA barcode identifications matched (>99 % sequence similarity) to DNA barcode records at BOLD repository. Specimens identified as phytoplasma positive (+) in final stage serial PCR using primer pairs (16F758F & M23Sr) or (fU5 & M23Sr). GenBank BLAST queries of phytoplasma PCR + sequences indicating closest matching 16Sr DNA sequence accession, percentage (%) similarity and accession identification.

specimen taxonomic and DNA barcode identifications					phytoplasma PCR+		GenBank BLAST query		
sample ID	field label	stage	taxonomic identification	DNA barcode identification	16F758F-M23Sr	fU5-M23Sr	accession	%	identification
ww17701	47	adult	*Austroagallia*	*Austroagallia torrida*					
ww17702	73	adult	*Austroagallia*	*Austroagallia torrida*					
ww17703	93	adult	*Austroagallia*	*Austroagallia torrida*					
ww17704	97	adult	*Austroagallia*	*Austroagallia torrida*					
ww17705	147	adult	*Orosius*	*Orosius argentatus*					
ww17707	199	adult	*Austroagallia*	*Austroagallia torrida*					
ww17708	41	adult	*Austroagallia*	*Austroagallia torrida*					
ww17709	200	adult	*Austroagallia*	*Austroagallia torrida*					
ww17710	201	adult	*Austroagallia*	*Austroagallia torrida*					
ww17711	202	adult	*Austroagallia*	*Austroagallia torrida*					
ww17712	205	adult	*Austroagallia*	*Austroagallia torrida*					
ww17713	211	adult	*Austroagallia*	*Austroagallia torrida*					
ww17714	215	adult	*Austroagallia*	*Austroagallia torrida*					
ww17715	217	adult	*Austroagallia*	*Austroagallia torrida*					
ww17716	218	adult	*Austroagallia*	*Austroagallia torrida*					
ww17717	222	adult	Miridae sp.	*Campylomma* sp.					
ww17718	227	adult	*Orosius*	*Orosius orientalis*					
ww17719	228	adult	*Austroagallia*	*Austroagallia torrida*	+		AM747040	>99	*Bacillus longiquaesitum*
ww17720	230	nymph	*Austroasca*	Typhlocybinae sp.					

ww17721	233	adult	*Austroagallia*	*Austroagallia torrida*					
ww17722	237	adult	*Austroagallia*	*Austroagallia torrida*					
ww17723	239	adult	*Austroasca*	Typhlocybinae sp.					
ww17724	245	adult	*Austroagallia*	*Austroagallia torrida*					
ww17725	246	adult	*Austroagallia*	*Austroagallia torrida*					
ww17726	251	adult	*Austroagallia*	*Austroagallia torrida*	+		GQ980248	>98	*Bacillus* sp. NBK47
ww17727	252	adult	*Austroagallia*	*Austroagallia torrida*	+	+	CP009920	>98	*Bacillus megaterium*
ww17728	253	adult	*Austroagallia*	*Austroagallia torrida*					
ww17729	257	adult	*Austroagallia*	*Austroagallia torrida*					
ww17730	258	adult	*Orosius*	*Orosius orientalis*					
ww17731	263	adult	*Austroagallia*	*Austroagallia torrida*					
ww17732	264	nymph	*Austroasca*	Typhlocybinae sp.					
ww17734	272	adult	*Austroagallia*	*Austroagallia torrida*					
ww17735	277	adult	*Austroagallia*	*Austroagallia torrida*					
ww17736	279	adult	*Austroagallia*	*Austroagallia torrida*					
ww17737	290	nymph	*Austroasca*	Typhlocybinae sp.					
ww17738	291	adult	*Austroagallia*	*Austroagallia torrida*					
ww17739	293	adult	*Austroasca*	Typhlocybinae sp.					
ww17740	295	adult	*Austroagallia*	*Austroagallia torrida*					
ww17741	297	adult	*Austroagallia*	*Austroagallia torrida*					
ww17742	299	adult	*Austroagallia*	*Austroagallia torrida*					
ww17743	300	adult	*Austroagallia*	*Austroagallia torrida*					
ww17744	302	adult	*Austroagallia*	*Austroagallia torrida*					
ww17745	303	adult	*Austroagallia*	*Austroagallia torrida*					
ww17746	304	adult	*Austroagallia*	*Austroagallia torrida*					
ww17748	310	adult	*Austroagallia*	*Austroagallia torrida*					
ww17749	314	adult	*Austroagallia*	*Austroagallia torrida*					
ww17750	320	adult	*Austroagallia*	*Austroagallia torrida*					
ww17751	321	adult	*Austroagallia*	*Austroagallia torrida*					
ww17753	328	adult	*Austroagallia*	*Austroagallia torrida*					

ww17754	330	adult	*Austroagallia*	*Austroagallia torrida*					
ww17755	333	adult	*Austroagallia*	*Austroagallia torrida*					
ww17756	338	nymph	*Austroasca*	Typhlocybinae sp.					
ww17757	339	adult	*Austroagallia*	*Austroagallia torrida*					
ww17758	347	adult	*Austroagallia*	*Austroagallia torrida*					
ww17759	348	adult	*Austroagallia*	*Austroagallia torrida*					
ww17760	354	adult	*Austroagallia*	*Austroagallia torrida*					
ww17762	361	adult	*Austroagallia*	*Austroagallia torrida*					
ww17763	363	adult	*Austroagallia*	*Austroagallia torrida*					
ww17764	377	adult	*Austroagallia*	*Austroagallia torrida*					
ww17766	381	adult	*Austroagallia*	*Austroagallia torrida*					
ww17767	383	adult	*Austroagallia*	*Austroagallia torrida*					
ww17768	388	adult	*Austroagallia*	*Austroagallia torrida*					
ww17769	389	adult	*Austroasca*	Typhlocybinae sp.					
ww17770	394	adult	*Austroasca*	Typhlocybinae sp.	+		KF364490	100	*Bacillus cereus*
ww17771	307	adult	*Austroagallia*	*Austroagallia torrida*					
ww17772	500	adult	*Austroagallia*	*Austroagallia torrida*	+		KC517358	>99	*Clostridium dakarense*
ww17774	401	adult	*Austroagallia*	*Austroagallia torrida*					
ww17775	414	adult	*Austroasca*	Typhlocybinae sp.					
ww17776	416	adult	*Austroagallia*	*Austroagallia torrida*	+	+	NR_041499	>99	*Flavisolibacter ginsengiterrae*
ww17777	419	adult	*Austroagallia*	*Austroagallia torrida*					
ww17778	422	adult	*Austroagallia*	*Austroagallia torrida*					
ww17780	431	nymph	*Austroasca*	Typhlocybinae sp.	+		HQ455822	>99	*Arthrobacter globiformis*
ww17781	439	adult	*Austroagallia*	*Austroagallia torrida*					
ww17782	440	adult	*Austroagallia*	*Austroagallia torrida*					
ww17783	441	adult	*Austroagallia*	*Austroagallia torrida*					
ww17784	444	adult	*Austroagallia*	*Austroagallia torrida*					
ww17785	455	adult	*Austroagallia*	*Austroagallia torrida*					
ww17786	459	adult	*Austroagallia*	*Austroagallia torrida*	+		NR_041499	>99	*Flavisolibacter ginsengiterrae*
ww17787	462	adult	*Austroagallia*	*Austroagallia torrida*	+		NR_041499	>99	*Flavisolibacter ginsengiterrae*

ww17788	464	adult	*Austroagallia*	*Austroagallia torrida*
ww17789	466	nymph	*Austroasca*	Typhlocybinae sp.
ww17790	467	adult	*Austroagallia*	*Austroagallia torrida*
ww17791	469	adult	*Austroagallia*	*Austroagallia torrida*
ww17792	472	nymph	*Austroasca*	Typhlocybinae sp.
ww17793	473	adult	*Austroagallia*	*Austroagallia torrida*
ww17794	468	adult	*Austroagallia*	*Austroagallia torrida*
ww18867	492	adult	*Austroagallia*	*Austroagallia torrida*
ww18870	476	adult	*Austroagallia*	*Austroagallia torrida*
ww18871	477	adult	*Austroagallia*	*Austroagallia torrida*
ww18872	479	adult	*Austroagallia*	*Austroagallia torrida*
ww18873	480	adult	*Austroagallia*	*Austroagallia torrida*
ww18874	482	adult	*Austroagallia*	*Austroagallia torrida*
ww18875	483	nymph	*Austroasca*	Typhlocybinae sp.
ww18876	486	adult	*Austroagallia*	*Austroagallia torrida*
ww18877	490	adult	*Austroagallia*	*Austroagallia torrida*

Supplementary Table S3: Phytoplasma 16SrDNA sequence accessions from GenBank, used in neighbour-joining distance analyses (Figure 1 & Supplementary Fig. 1). Accessions in lucerne as listed.

GenBank accession	accession description	in lucerne
AB010425.2	Candidatus Phytoplasma japonicum	
AB052873.1	Candidatus Phytoplasma oryzae	
AB052876.1	Phytoplasma sp. JWB-G1	
AB054986.1	Candidatus Phytoplasma castaneae	
AB247462.1	Candidatus Phytoplasma aurantifolia	
AB259169.1	Alfalfa witches'-broom phytoplasma	Yes
AB279597.1	Candidatus Phytoplasma trifolii	
AB667970.1	Loofah witches'-broom phytoplasma	
AB690303.1	Aster virescence phytoplasma	
AB741637.1	Pigeon pea witches'-broom phytoplasma	
AF036354.1	'*Fragaria multicipita'* phytoplasma	
AF060875.1	Virginia grapevine yellows phytoplasma VGYIII	
AF086621.2	Loofah witches'-broom phytoplasma	
AF092209.1	Ash yellows phytoplasma	
AF147706.1	Chayote witches' broom phytoplasma ChWBIII strain	
AF147708.1	Hibiscus witches'-broom phytoplasma strain HibWB26	
AF177384.1	Alfalfa stunt phytoplasma	Yes
AF190223.1	Poinsettia branch-inducing phytoplasma	
AF190226.1	Walnut witches' broom phytoplasma	
AF190227.1	Walnut witches' broom phytoplasma	
AF190228.1	Spiraea stunt phytoplasma	
AF222065.1	Clover phyllody phytoplasma strain CPh	
AF248956.1	Loofah witches'-broom phytoplasma	
AF248957.1	Pigeon pea witches'-broom phytoplasma	
AF248960.1	Mexican periwinkle virescence phytoplasma	
AF268405.1	Aster yellows phytoplasma O isolate 98UW166B	
AF268895.1	Elm phytoplasma 'Arlington Heights'	
AF274876.1	Strawberry leafy fruit phytoplasma	
AF302841.1	Black raspberry witches'-broom phytoplasma clone BRWB7	
AF353090.1	Loofah witches'-broom phytoplasma strain LfWB 1	
AF370119.1	Dandelion virescence phytoplasma	
AF370120.1	Dandelion virescence phytoplasma	
AF373105.1	Cirsium white leaf phytoplasma	
AF373106.1	Cirsium white leaf phytoplasma	
AF409069.1	Clover proliferation phytoplasma strain EY-IL 2	
AF409070.1	Clover proliferation phytoplasma strain EY-IL 1	
AF438413.1	Omani alfalfa witches'-broom phytoplasma	Yes

AF498307.1	Coconut lethal yellowing phytoplasma strain Jamaica LYJ-C8	
AF503568.1	Aster yellows phytoplasma I-P	
AF510724.1	Milkweed yellows phytoplasma	
AF515636.1	Candidatus Phytoplasma phoenicium strain A4	
AF515637.1	Candidatus Phytoplasma phoenicium strain 21	
AF533231.1	Western X phytoplasma	
AJ289192.2	Stylosanthes little leaf phytoplasma	present study
AJ289193.2	Sweet potato little leaf phytoplasma	
AJ310849.2	Phytoplasma sp.	
AJ315965.1	Australian lucerne yellows phytoplasma	Yes
AJ542541.1	Candidatus Phytoplasma mali	
AJ542543.1	Candidatus Phytoplasma pyri	
AJ542544.1	Candidatus Phytoplasma prunorum	
AJ548787.2	Flavescence doree phytoplasma partial	
AJ550984.1	Bermuda grass white leaf phytoplasma	
AY034608.1	Erigeron witches'-broom phytoplasma	
AY102275.1	Strawberry phylloid fruit phytoplasma	
AY135523.1	'*Allocasuarina muelleriana*' phytoplasma clone 2	
AY135524.1	'*Allocasuarina muelleriana*' phytoplasma clone 3	
AY147038.1	Argentinian alfalfa witches'-broom phytoplasma	Yes
AY169322.1	Alfalfa witches'-broom phytoplasma	Yes
AY169323.1	Alfalfa witches'-broom phytoplasma	Yes
AY180957.1	Aster yellows phytoplasma strain AV2192	
AY197642.1	Alder yellows phytoplasma strain ALY882	
AY197648.1	Rubus stunt phytoplasma strain RUS	
AY197655.1	Elm yellows phytoplasma strain EY1	
AY265205.1	Aster yellows phytoplasma strain IOWB	
AY265206.1	Aster yellows phytoplasma strain PaWB	
AY265209.1	Aster yellows phytoplasma strain AVUT	
AY265211.1	Aster yellows phytoplasma strain ACLR-AY	
AY265213.1	Blueberry stunt phytoplasma strain BBS3	
AY390261.1	Candidatus Phytoplasma trifolii	
AY725211.2	Bolivia alfalfa phytoplasma	Yes
AY725228.1	Candidatus Phytoplasma graminis	
AY725234.1	Candidatus Phytoplasma caricae	
DQ086423.1	Candidatus Phytoplasma fragariae	
DQ174122.1	Candidatus Phytoplasma americanum strain PPT12-NE	
DQ233655.1	Fars(Iran) alfalfa witches'-broom phytoplasma	Yes
DQ233656.1	Yazd(Iran) alfalfa witches'-broom phytoplasma	Yes
DQ305982.2	Alfalfa phytoplasma	Yes
DQ786394.1	Candidatus Phytoplasma australiense	Yes

DQ826446.1	Lucerne witches'-broom phytoplasma	Yes
EF186821.1	Lucerne virescence phytoplasma strain LUM	Yes
EF186827.1	Cotton phyllody phytoplasma strain CoP	
EF193356.1	Italian alfalfa witches'-broom phytoplasma	Yes
EF193358.1	Sunn hemp witches'-broom phytoplasma	
EF193359.1	Tomato big bud phytoplasma	
EF193360.1	Alfalfa witches'-broom phytoplasma	Yes
EF199549.2	Candidatus Phytoplasma lycopersici isolate Santa Cruz	
EF666051.1	Candidatus Phytoplasma omanense strain IM-1	
EU346761.1	Cassava frogskin disease phytoplasma strain FSDY15	
EU371934.2	Malaysian periwinkle virescence phytoplasma strain MaPV	
FJ231728.1	Cherry decline phytoplasma	
FJ432664.1	Salt cedar witches'-broom phytoplasma strain SCWB1	
FJ788514.1	Alfalfa witches'-broom phytoplasma Urmia	Yes
FJ943262.1	Candidatus Phytoplasma australiense strain NZ09156	
GU075849.1	'*Medicago sativa'* phytoplasma isolate Mesa09	Yes
GU289675.1	Alfalfa stunt phytoplasma strain AlfS-L	Yes
HQ225630.1	Soybean stunt phytoplasma isolate SoyST1c1	
HQ404357.1	*Bituminaria bituminosa* witches'-broom phytoplasma NA-PH1-1	
HQ436488.1	Chile pepper phytoplasma strain Brote Grande NM	
JF508514.1	Sesame phyllody phytoplasma strain Seph2	
JF508517.1	Cucumber phyllody phytoplasma strain Cuph2	
JN652666.1	Candidatus Phytoplasma australiense isolate 840	
JN860711.1	Bushehr(Iran) alfalfa witches'-broom phytoplasma B1	Yes
JQ045568.1	*'Medicago sativa*' yellowing phytoplasma isolate Alhasa clone 2	Yes
JQ067649.1	Sweet potato little leaf phytoplasma strain V4	
JQ343221.1	Alfalfa witches'-broom phytoplasma AWB-YL	Yes
JQ412094.2	Iranian alfalfa phytoplasma AF16	Yes
JQ412095.2	Iranian alfalfa phytoplasma KER	Yes
JQ412096.1	Iranian alfalfa phytoplasma T22	Yes
JQ412097.1	Iranian alfalfa phytoplasma X11	Yes
JQ412099.1	Iranian alfalfa phytoplasma K14	Yes
JQ412100.1	Iranian alfalfa phytoplasma M21	Yes
JQ579448.1	Iranian alfalfa phytoplasma U42	Yes
JQ579449.2	Iranian alfalfa phytoplasma W21	Yes
JQ951959.1	Alfalfa yellows phytoplasma isolate L41	Yes
JQ951960.1	Candidatus Phytoplasma solani isolate L42	Yes
JX272797.1	Australian lucerne yellows phytoplasma strain LYSP	Yes
JX861231.1	Australian lucerne yellows phytoplasma strain LYSP-E2	Yes
JX861232.1	Australian lucerne yellows phytoplasma strain LYSP-E6	Yes

JX861233.1	Australian lucerne yellows phytoplasma strain LYSP-D7	Yes
JX861234.1	Australian lucerne yellows phytoplasma strain LYSP-D8	Yes
KC261849.1	Alfalfa witches'-broom phytoplasma strain Nik21	Yes
KC261850.1	Alfalfa witches'-broom phytoplasma strain Nik22	Yes
KC261851.1	Alfalfa witches'-broom phytoplasma strain Nik23	Yes
KC508646.1	Soybean witches'-broom phytoplasma isolate NDT&HTT7879	
KF178706.1	Candidatus Phytoplasma trifolii	
KF607104.1	Fars(Iran) alfalfa witches'-broom phytoplasma	Yes
KF607106.1	Yazd(Iran) alfalfa witches'-broom phytoplasma	Yes
KP864671.1	Candidatus Phytoplasma trifolii strain Rus-361Fc	Yes
KR072666.1	Columbia Basin potato purple top phytoplasma strain WA1	
KT750060.1	Iranian alfalfa witches'-broom phytoplasma isolate Mes 95	Yes
KT763371.1	Iranian alfalfa witches'-broom phytoplasma isolate Mes 37	Yes
KT763372.1	Iranian alfalfa witches'-broom phytoplasma isolate Mes 45	Yes
KT763373.1	Iranian alfalfa witches'-broom phytoplasma isolate Mes 35	Yes
KT781662.1	Iranian alfalfa witches'-broom phytoplasma isolate Mes 30	Yes
KT932705.1	Lucerne witches'-broom phytoplasma	Yes
KT943963.1	Alfalfa witches'-broom phytoplasma clone Mes 98	Yes
KT943964.1	Alfalfa witches'-broom phytoplasma clone Mes 38	Yes
KT943965.1	Alfalfa witches'-broom phytoplasma clone Mes 63	Yes
KT943966.1	Alfalfa witches'-broom phytoplasma clone Mes 125	Yes
KT943967.1	Alfalfa witches'-broom phytoplasma clone Mes 1	Yes
KT943970.1	Alfalfa witches'-broom phytoplasma clone Mes 20	Yes
KT943971.1	Alfalfa witches'-broom phytoplasma clone Mes 64, 66	Yes
KU170535.1	'*Vigna unguiculata subsp. sesquipedalis*' phytoplasma UV-3	
KU240021.1	Alfalfa witches'-broom phytoplasma Mes40	Yes
L33760.1	MPLDRRC Tomato big bug mycoplasma-like organism	
L33765.1	MPLDRRI Peanut witches'-broom mycoplasma-like organism	
L76865.1	CDTRG16R Australian grapevine yellows phytoplasma	
M30790.1	MPLRRD Oenothera phytoplasma	
MOU18753.2	Yucatan coconut lethal decline phytoplasma	
NC007716.1	Aster yellows witches'-broom phytoplasma AYWB	
U15442.1	MOU15442 Candidatus Phytoplasma aurantifolia	
U96614.1	PSU96614 Phytoplasma sp. STRAWB1	
U96616.1	PSU96616 Phytoplasma sp. STRAWB2	
X76427.1	Mollicutes (from C.anuum to C.roseus)	

X76429.1	Mollicutes (from C.roseus)	
X76432.1	Mollicutes (from S.officinarum)	
X76560.1	Mycoplasma (MLO;FD) transmitted from V.vinifera to V.faba	
X80117.1	Phytoplasma sp.	
X83432.1	Mollicutes sp.	
X92869.1	Phytoplasma sp.	
Y16390.1	Italian alfalfa witches-broom phytoplasma	Yes
Y16393.1	*Picris echiodes* phyllody phytoplasma	
Y17055.1	Phytoplasma sp.(strain StLL)	

Supplementary Figure 1: Uncollapsed neighbour-joining distance tree of 16S rDNA phytoplasma sequence accessions (N=163) and sequences of nine phytoplasma-positive lucerne from Forbes, Australia. Scale bar equals 1% equal weighted sequence difference. Cluster node supports > 70 % (10,000 bootstrap replicates) as indicated. Tip labels indicate GenBank accession numbers and description; Forbes specimen sequences simply labelled with “ww” identification number (refer Sup. Table S1).

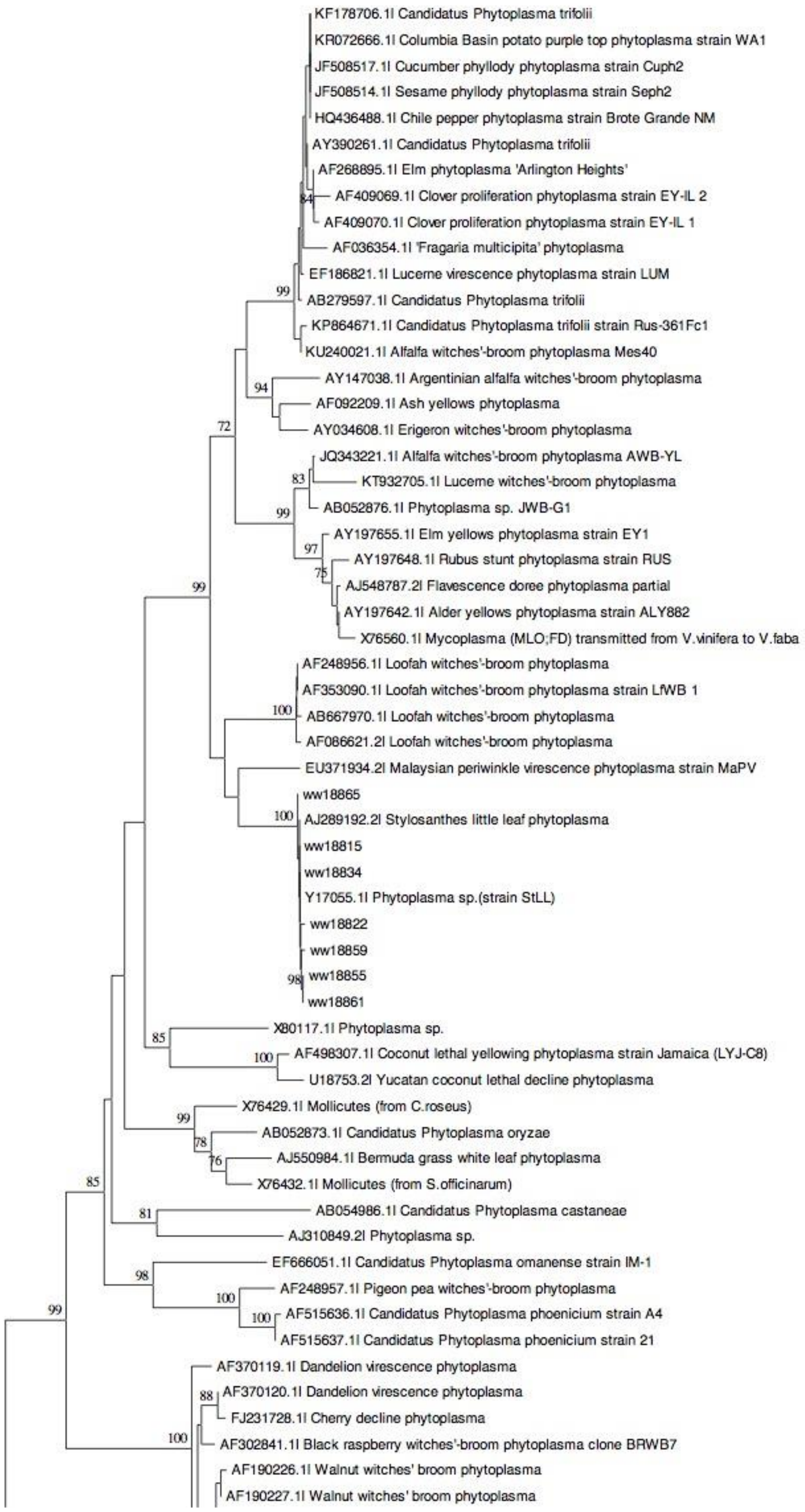
KF178706.1l Candidatus Phytoplasma trifolii
KR072666.1l Columbia Basin potato purple top phytoplasma strain WA1
JF508517.1l Cucumber phyllody phytoplasma strain Cuph2
JF508514.1l Sesame phyllody phytoplasma strain Seph2
HQ436488.1l Chile pepper phytoplasma strain Brote Grande NM
AY390261.1l Candidatus Phytoplasma trifolii
AF268895.1l Elm phytoplasma 'Arlington Heights'
AF409069.1l Clover proliferation phytoplasma strain EY-IL 2
AF409070.1l Clover proliferation phytoplasma strain EY-IL 1
AF036354.1l 'Fragaria multicipita' phytoplasma
EF186821.1l Lucerne virescence phytoplasma strain LUM
AB279597.1l Candidatus Phytoplasma trifolii
KP864671.1l Candidatus Phytoplasma trifolii strain Rus-361Fc1
KU240021.1l Alfalfa witches'-broom phytoplasma Mes40
AY147038.1l Argentinian alfalfa witches'-broom phytoplasma
AF092209.1l Ash yellows phytoplasma
AY034608.1l Erigeron witches'-broom phytoplasma
JQ343221.1l Alfalfa witches'-broom phytoplasma AWB-YL
KT932705.1l Lucerne witches'-broom phytoplasma
AB052876.1l Phytoplasma sp. JWB-G1
AY197655.1l Elm yellows phytoplasma strain EY1
AY197648.1l Rubus stunt phytoplasma strain RUS
AJ548787.2l Flavescence doree phytoplasma partial
AY197642.1l Alder yellows phytoplasma strain ALY882
X76560.1l Mycoplasma (MLO;FD) transmitted from V.vinifera to V.faba
AF248956.1l Loofah witches'-broom phytoplasma
AF353090.1l Loofah witches'-broom phytoplasma strain LfWB 1
AB667970.1l Loofah witches'-broom phytoplasma
AF086621.2l Loofah witches'-broom phytoplasma
EU371934.2l Malaysian periwinkle virescence phytoplasma strain MaPV
ww18865
AJ289192.2l Stylosanthes little leaf phytoplasma
ww18815
ww18834
Y17055.1l Phytoplasma sp.(strain StLL)
ww18822
ww18859
ww18855
ww18861
X80117.1l Phytoplasma sp.
AF498307.1l Coconut lethal yellowing phytoplasma strain Jamaica (LYJ-C8)
U18753.2l Yucatan coconut lethal decline phytoplasma
X76429.1l Mollicutes (from C.roseus)
AB052873.1l Candidatus Phytoplasma oryzae
AJ550984.1l Bermuda grass white leaf phytoplasma
X76432.1l Mollicutes (from S.officinarum)
AB054986.1l Candidatus Phytoplasma castaneae
AJ310849.2l Phytoplasma sp.
EF666051.1l Candidatus Phytoplasma omanense strain IM-1
AF248957.1l Pigeon pea witches'-broom phytoplasma
AF515636.1l Candidatus Phytoplasma phoenicium strain A4
AF515637.1l Candidatus Phytoplasma phoenicium strain 21
AF370119.1l Dandelion virescence phytoplasma
AF370120.1l Dandelion virescence phytoplasma
FJ231728.1l Cherry decline phytoplasma
AF302841.1l Black raspberry witches'-broom phytoplasma clone BRWB7
AF190226.1l Walnut witches' broom phytoplasma
AF190227.1l Walnut witches' broom phytoplasma
84
99
94
72
83
99
97
75
99
100
100
98
85
100
99
78
76
85
81
98
100
100
99
88
100

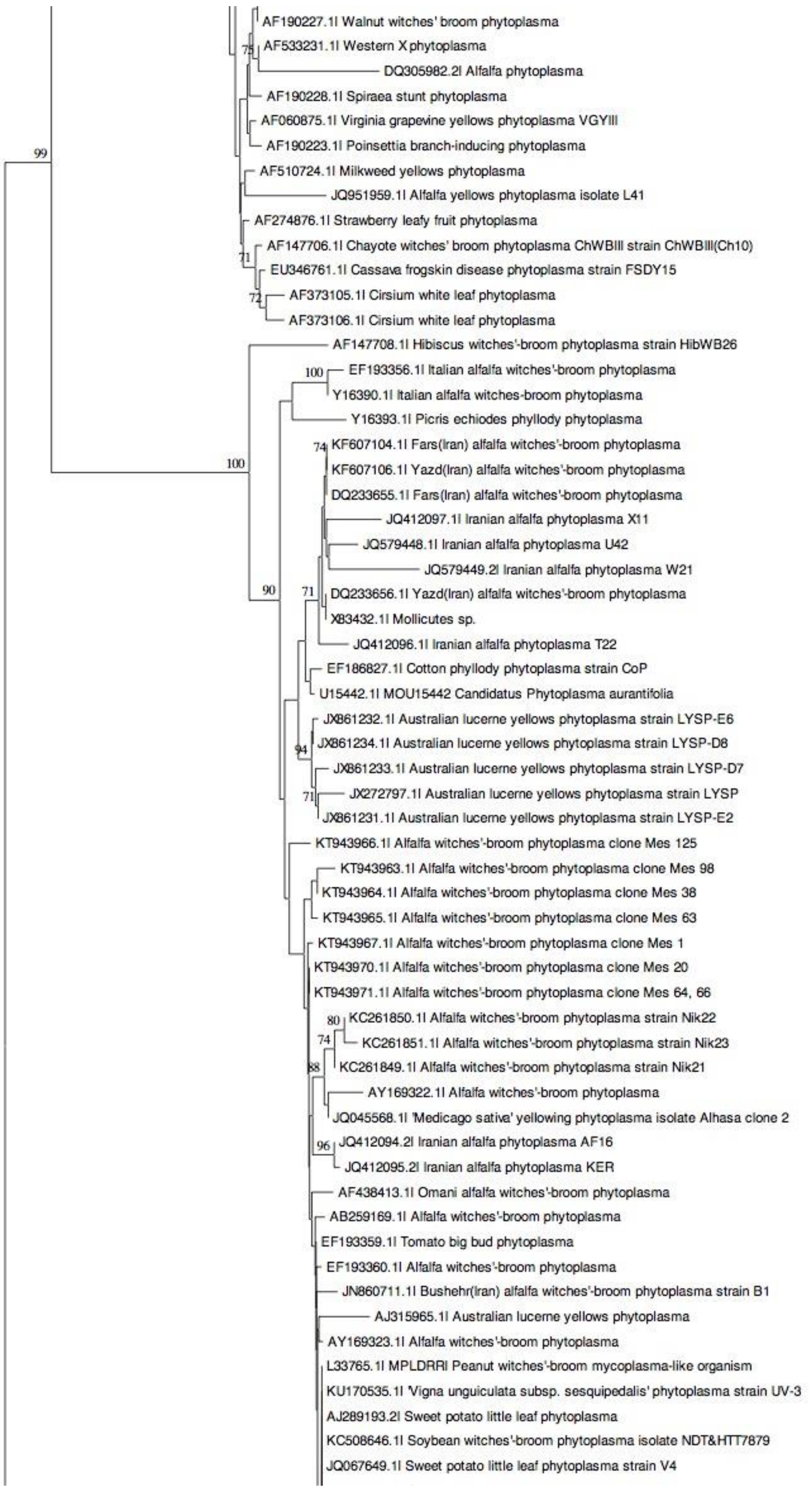
99
AF190227.1| Walnut witches' broom phytoplasma
75
AF533231.1| Western X phytoplasma
DQ305982.2| Alfalfa phytoplasma
AF190228.1| Spiraea stunt phytoplasma
AF060875.1| Virginia grapevine yellows phytoplasma VGYIII
AF190223.1| Poinsettia branch-inducing phytoplasma
AF510724.1| Milkweed yellows phytoplasma
JQ951959.1| Alfalfa yellows phytoplasma isolate L41
AF274876.1| Strawberry leafy fruit phytoplasma
AF147706.1| Chayote witches' broom phytoplasma ChWBIII strain ChWBIII(Ch10)
71
EU346761.1| Cassava frogskin disease phytoplasma strain FSDY15
72
AF373105.1| Cirsium white leaf phytoplasma
AF373106.1| Cirsium white leaf phytoplasma
AF147708.1| Hibiscus witches'-broom phytoplasma strain HibWB26
100
EF193356.1| Italian alfalfa witches'-broom phytoplasma
Y16390.1| Italian alfalfa witches-broom phytoplasma
Y16393.1| Picris echiodes phyllody phytoplasma
100
74
KF607104.1| Fars(Iran) alfalfa witches'-broom phytoplasma
KF607106.1| Yazd(Iran) alfalfa witches'-broom phytoplasma
DQ233655.1| Fars(Iran) alfalfa witches'-broom phytoplasma
JQ412097.1| Iranian alfalfa phytoplasma X11
JQ579448.1| Iranian alfalfa phytoplasma U42
JQ579449.2| Iranian alfalfa phytoplasma W21
90
71
DQ233656.1| Yazd(Iran) alfalfa witches'-broom phytoplasma
X83432.1| Mollicutes sp.
JQ412096.1| Iranian alfalfa phytoplasma T22
EF186827.1| Cotton phyllody phytoplasma strain CoP
U15442.1| MOU15442 Candidatus Phytoplasma aurantifolia
JX861232.1| Australian lucerne yellows phytoplasma strain LYSP-E6
94
JX861234.1| Australian lucerne yellows phytoplasma strain LYSP-D8
JX861233.1| Australian lucerne yellows phytoplasma strain LYSP-D7
71
JX272797.1| Australian lucerne yellows phytoplasma strain LYSP
JX861231.1| Australian lucerne yellows phytoplasma strain LYSP-E2
KT943966.1| Alfalfa witches'-broom phytoplasma clone Mes 125
KT943963.1| Alfalfa witches'-broom phytoplasma clone Mes 98
KT943964.1| Alfalfa witches'-broom phytoplasma clone Mes 38
KT943965.1| Alfalfa witches'-broom phytoplasma clone Mes 63
KT943967.1| Alfalfa witches'-broom phytoplasma clone Mes 1
KT943970.1| Alfalfa witches'-broom phytoplasma clone Mes 20
KT943971.1| Alfalfa witches'-broom phytoplasma clone Mes 64, 66
80
KC261850.1| Alfalfa witches'-broom phytoplasma strain Nik22
74
KC261851.1| Alfalfa witches'-broom phytoplasma strain Nik23
88
KC261849.1| Alfalfa witches'-broom phytoplasma strain Nik21
AY169322.1| Alfalfa witches'-broom phytoplasma
JQ045568.1| 'Medicago sativa' yellowing phytoplasma isolate Alhasa clone 2
96
JQ412094.2| Iranian alfalfa phytoplasma AF16
JQ412095.2| Iranian alfalfa phytoplasma KER
AF438413.1| Omani alfalfa witches'-broom phytoplasma
AB259169.1| Alfalfa witches'-broom phytoplasma
EF193359.1| Tomato big bud phytoplasma
EF193360.1| Alfalfa witches'-broom phytoplasma
JN860711.1| Bushehr(Iran) alfalfa witches'-broom phytoplasma strain B1
AJ315965.1| Australian lucerne yellows phytoplasma
AY169323.1| Alfalfa witches'-broom phytoplasma
L33765.1| MPLDRRI Peanut witches'-broom mycoplasma-like organism
KU170535.1| 'Vigna unguiculata subsp. sesquipedalis' phytoplasma strain UV-3
AJ289193.2| Sweet potato little leaf phytoplasma
KC508646.1| Soybean witches'-broom phytoplasma isolate NDT&HTT7879
JQ067649.1| Sweet potato little leaf phytoplasma strain V4

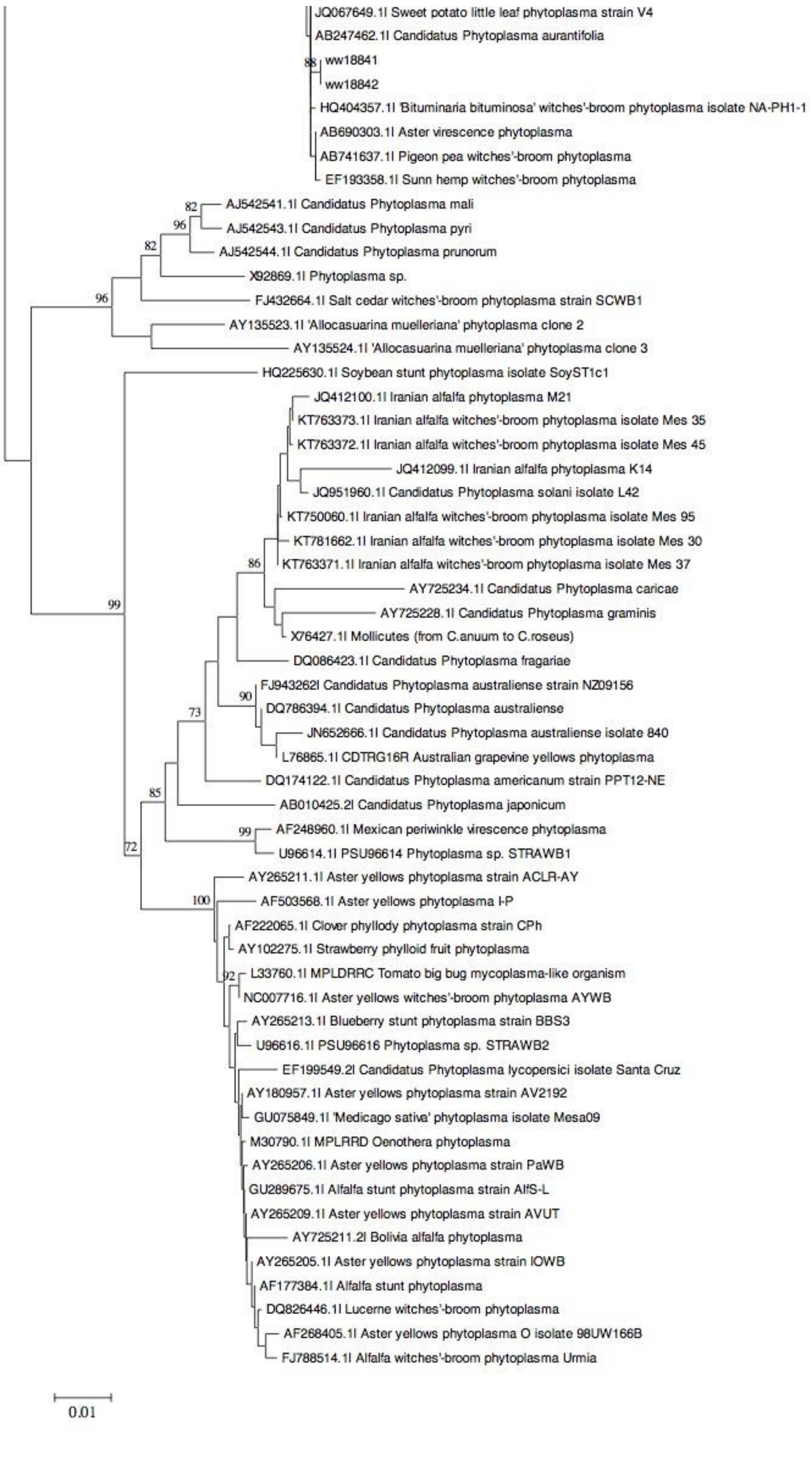

JQ067649.1l Sweet potato little leaf phytoplasma strain V4
AB247462.1l Candidatus Phytoplasma aurantifolia
ww18841
ww18842
HQ404357.1l 'Bituminaria bituminosa' witches'-broom phytoplasma isolate NA-PH1-1
AB690303.1l Aster virescence phytoplasma
AB741637.1l Pigeon pea witches'-broom phytoplasma
EF193358.1l Sunn hemp witches'-broom phytoplasma
AJ542541.1l Candidatus Phytoplasma mali
AJ542543.1l Candidatus Phytoplasma pyri
AJ542544.1l Candidatus Phytoplasma prunorum
X92869.1l Phytoplasma sp.
FJ432664.1l Salt cedar witches'-broom phytoplasma strain SCWB1
AY135523.1l 'Allocasuarina muelleriana' phytoplasma clone 2
AY135524.1l 'Allocasuarina muelleriana' phytoplasma clone 3
HQ225630.1l Soybean stunt phytoplasma isolate SoyST1c1
JQ412100.1l Iranian alfalfa phytoplasma M21
KT763373.1l Iranian alfalfa witches'-broom phytoplasma isolate Mes 35
KT763372.1l Iranian alfalfa witches'-broom phytoplasma isolate Mes 45
JQ412099.1l Iranian alfalfa phytoplasma K14
JQ951960.1l Candidatus Phytoplasma solani isolate L42
KT750060.1l Iranian alfalfa witches'-broom phytoplasma isolate Mes 95
KT781662.1l Iranian alfalfa witches'-broom phytoplasma isolate Mes 30
KT763371.1l Iranian alfalfa witches'-broom phytoplasma isolate Mes 37
AY725234.1l Candidatus Phytoplasma caricae
AY725228.1l Candidatus Phytoplasma graminis
X76427.1l Mollicutes (from C.anuum to C.roseus)
DQ086423.1l Candidatus Phytoplasma fragariae
FJ943262l Candidatus Phytoplasma australiense strain NZ09156
DQ786394.1l Candidatus Phytoplasma australiense
JN652666.1l Candidatus Phytoplasma australiense isolate 840
L76865.1l CDTRG16R Australian grapevine yellows phytoplasma
DQ174122.1l Candidatus Phytoplasma americanum strain PPT12-NE
AB010425.2l Candidatus Phytoplasma japonicum
AF248960.1l Mexican periwinkle virescence phytoplasma
U96614.1l PSU96614 Phytoplasma sp. STRAWB1
AY265211.1l Aster yellows phytoplasma strain ACLR-AY
AF503568.1l Aster yellows phytoplasma I-P
AF222065.1l Clover phyllody phytoplasma strain CPh
AY102275.1l Strawberry phylloid fruit phytoplasma
L33760.1l MPLDRRC Tomato big bug mycoplasma-like organism
NC007716.1l Aster yellows witches'-broom phytoplasma AYWB
AY265213.1l Blueberry stunt phytoplasma strain BBS3
U96616.1l PSU96616 Phytoplasma sp. STRAWB2
EF199549.2l Candidatus Phytoplasma lycopersici isolate Santa Cruz
AY180957.1l Aster yellows phytoplasma strain AV2192
GU075849.1l 'Medicago sativa' phytoplasma isolate Mesa09
M30790.1l MPLRRD Oenothera phytoplasma
AY265206.1l Aster yellows phytoplasma strain PaWB
GU289675.1l Alfalfa stunt phytoplasma strain AlfS-L
AY265209.1l Aster yellows phytoplasma strain AVUT
AY725211.2l Bolivia alfalfa phytoplasma
AY265205.1l Aster yellows phytoplasma strain IOWB
AF177384.1l Alfalfa stunt phytoplasma
DQ826446.1l Lucerne witches'-broom phytoplasma
AF268405.1l Aster yellows phytoplasma O isolate 98UW166B
FJ788514.1l Alfalfa witches'-broom phytoplasma Urmia
0.01
